# Microduplication of 3p26.3 Implicated in Cognitive Development

**DOI:** 10.1155/2014/295359

**Published:** 2014-02-13

**Authors:** Leah Te Weehi, Raj Maikoo, Adrian Mc Cormack, Roberto Mazzaschi, Fern Ashton, Liangtao Zhang, Alice M. George, Donald R. Love

**Affiliations:** ^1^Diagnostic Genetics, LabPLUS, Auckland City Hospital, P.O. Box 110031, Auckland 1148, New Zealand; ^2^Pediatrics Department, Middlemore Hospital, Private Bag 93311, Auckland 1640, New Zealand; ^3^School of Biological Sciences, University of Auckland, Private Bag 92019, Auckland 1142, New Zealand

## Abstract

We report here a 34-month-old boy with global developmental delay referred for molecular karyotyping and fragile X studies. Molecular karyotype analysis revealed a microduplication in the 3p26.3 region involving part of the *CHL1* and *CNTN6* genes. Several deletions, one translocation, and one duplication have previously been described in this region of chromosome 3. The *CHL1* gene has been proposed as a dosage-sensitive gene with a central role in cognitive development, and so the microduplication reported here appears to be implicated in our patient's phenotype.

## 1. Introduction

**Figure 1:**
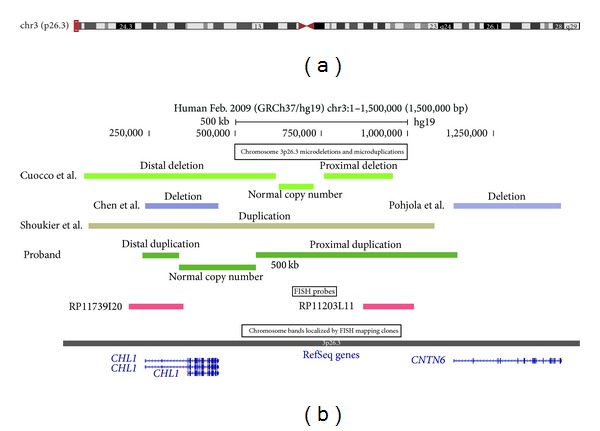
Schematic of chromosome 3p26.3 showing microdeletions and microduplications. (a) Shows the ideogram of chromosome 3, together with the region encompassing microdeletions and microduplications (red box). (b) Shows the location and extent of the microdeletions and microduplications detected in the proband reported here and other cases reported in the literature [6–8, 13], BAC probes used in the FISH studies, and RefSeq genes that lie within this region of chromosome 3. These graphics were taken from the UCSC genome browser [14].

Anomalies of the distal portion of the short arm of chromosome 3 are rare and not yet fully understood. The most well-characterised anomalies are deletions. For the most part, they occur *de novo*, although a few familial cases have been reported [1–8]. These deletions range from one to several megabases, but the extent of the deletion does not correlate with phenotypic severity. The clinical syndrome includes intellectual disability, low birth weight, micro- and trigonocephaly, and characteristic facial features such as ptosis, telecanthus, downslanting palpebral fissures, and micrognathia. Many genes have been implicated to play a role: *CRBN* and *CNTN4* have been suggested to cause typical 3p deletion syndrome [9, 10], and the *CHL1* gene has been proposed to play an additional role in cognitive impairment [8, 11–13]. The involvement of the *CHL1* gene has been reported in four previous case studies: three with deletions confined to 3p26.3 [6–8], including only the *CHL1* gene, a translocation [12], and one novel microduplication [13] (Figure 1). In these cases the growth abnormalities and typical facial features of 3p deletion syndrome were absent. Nonspecific intellectual disability was the main trait.

Interestingly, the previously reported 3p26.3 microduplication case manifested similar clinical features to those patients carrying a *CHL1* gene deletion, namely, nonspecific intellectual disability and epilepsy [13]. Epilepsy was also present in one child with a submicroscopic 3p26.3 noncontiguous terminal deletion containing only the *CHL1* gene [6].

We have identified a second case involving 3p26.3 microduplication that encompasses part of the *CHL1* gene as well as the *CNTN6* gene. Our case presents with motor and speech developmental delay and some autistic features.

## 2. Materials and Methods

Genomic deoxyribonucleic acid (DNA) was isolated from the peripheral blood using the Gentra Puregene Blood Kit (QIAGEN Genomics, Bothell, Washington, USA) according to the manufacturer's instructions. 0.1 micrograms of genomic DNA was labelled using the Affymetrix Cytogenetics Reagent Kit and labelled DNA was applied to an Affymetrix Whole-Genome 750K chip according to the manufacturer's instructions (Affymetrix Inc., CA, USA). The array was scanned and the data analysed using the Affymetrix Chromosome Analysis Suite (ChAS; version 1.0.1) and interpreted with the aid of the UCSC genome browser (http://genome.ucsc.edu/) [14]. All genomic coordinates were taken from the February 2009 (hg19) human reference sequence (NCBI Build 37), and gene and Online Mendelian Inheritance in Man (OMIM) references were from RefSeq and OMIM entries, respectively [14, 15].

A peripheral blood sample was collected in heparin from the proband and cultured according to standard cytogenetic protocols. Based on the duplicated region revealed by molecular karyotype analysis, two 3p26.3 locus-specific Bacterial Artificial Chromosome (BAC) probes, RP11-739I20 (SpG) (hg19 coordinates: chr3:190,761-349,109) and RP11-203L11 (SpO) (hg19 coordinates: chr3:871,241-1,019,847), were chosen from the Human BAC DNA library-32K set. They were labelled with green and orange fluorescent dyes, respectively. The Fluorescence *in situ* hybridisation (FISH) method followed the procedure of Pinkel et al. [16] with some modifications. Codenaturation was achieved by placing the slides into a thermal cycler (PTC-200) preheated to 87°C for 2 minutes. The slides were hybridized overnight in a humidified chamber at 37°C. The following day, the slides were subjected to a stringent wash in 0.4X SSC at 74°C for 2 minutes followed by 2X SSC at room temperature for 1 minute. After the slides were air-dried, 8 *μ*L of mounting medium (Vectashield) was applied to each of the slides. FISH was performed on 10 metaphase cells. The images were captured using a MetaSystems ISIS imaging system on a Zeiss AXIO Imager, M1 fluorescence microscope with sequential DAPI, and spectral green and spectral orange filter settings.

## 3. Case Report

The patient is the second male child of healthy nonconsanguineous parents. There is no family history of syndromes or developmental delay. His four-year-old brother is normal. He was born at 38 weeks gestation via emergency caesarean section for breech position. He had a birth weight of 2930 g (2nd to 9th percentile) [17]. There were no antenatal problems or intrapartum complications. He was born with syndactyly of the right fourth and fifth metacarpals with shortened little finger and a hypoplastic right thumb. Careful examination revealed partial syndactyly of his second and third toes on both feet. He underwent surgical correction of his right hand syndactyly (division of synostosis and interlay of dermal fat graft) at almost 15 months and is awaiting surgery for his thumb.

The patient was referred to paediatric services at approximately 15 months of age for gross motor developmental concerns. These were first noticed at 5 months of age. He was able to sit with support at 10 months and independently at 13 months of age. By 15 months he was still not pulling himself to stand or crawling. Examination at 15 months showed a weight of 10.05 kg (10th to 25th percentile), length of 76.6 cm (25th percentile), and a head circumference of 47.5 cm (50th to 75th percentile). Prior to this, his growth parameters were reported to be tracking along the 10th to 25th percentiles. He had intact cranial nerves and normal neurology in his upper limbs. Lower limb examination revealed mild pes cavus and increased tone especially around the Achilles tendon. Both the cavus and tone were more significant on the left side. Knee and ankle jerks were hyperreflexic but plantars were both downgoing. The rest of the examination was unremarkable. The dominant finding was lower limb spasticity.

When seen again at 25 months of age, he was noted to be still delayed in the gross motor domain but also delayed in other domains. Fine motor skills and receptive and expressive language skills were at 18 months developmental stage and social and self-care skills at the 12 months stage. He was observed to have a large prominent forehead and button-shaped nose but no gross dysmorphic features. None of the characteristic facial features of the 3p deletions syndrome were observed. Features he demonstrated that were suggestive of autistic spectrum disorder were repetitive activities, preoccupation with spinning wheels, resistance to changes in routine, and heightened sensitivity to people touching his legs. Overall, however, he was not thought to have an autistic spectrum disorder.

He was referred to a speech language therapist, physiotherapist, and a neurodevelopmental therapist. Under these health professionals he made significant progress over several months. At 28 months of age, both receptive and expressive language domains were mildly delayed to the level of 22–24 months. His gross motor skills were similarly only mildly delayed. By 32 months he was able to mobilise 15 metres and transfer to standing through half-kneeling with one arm for support. The lower limb spasticity improved.

He underwent general investigations for developmental delay. The MRI scan of his brain was unremarkable and his creatinine kinase level was 35 U/L (normal age-specific range 30–150) [18]. Fragile X testing involved PCR amplification and then fluorescence-based capillary electrophoresis to determine the number of CGG repeats within the *FMR-1* gene [19]. He was found to have a normal CGG repeat length of 30.

Molecular karyotype analysis revealed a 913 kb region of allelic imbalance involving the 3p26.3 region (chr3:231,390-1,144,815; hg19 coordinates; data not shown). This allelic imbalance was complicated in that it comprised approximately 220 kb of neutral copy number flanked by regions with a copy number change consistent with duplication. These flanking regions, corresponding to chr3:231,390-336,272 and chr3:559,569-1,144,815, encompass the amino terminal regions of the *CHL1* and *CNTN6* genes, respectively (Figure 1).

**Figure 2:**
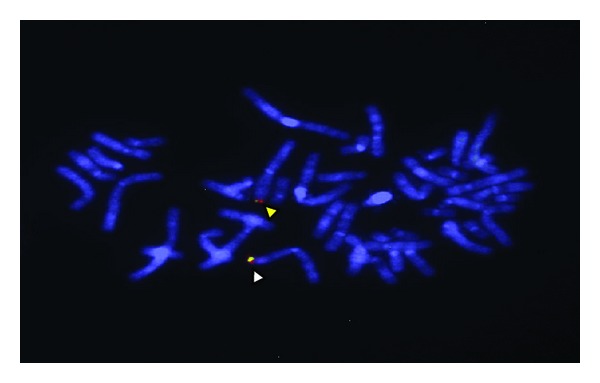
FISH analysis of metaphase cells using two BAC probes. The fluorescent signals identify the two homologues of chromosome 3 in cells of the proband. The orange and green signals (indicated by the yellow arrow head) show hybridisation of the BAC clones RP11-203L11 and RP11-739I20, respectively, to their normal sequences. The lower homolog shows a yellow signal, created from the combination of orange and green signals (indicated by a white arrow head), which is consistent with two tandem duplications on the same homologue.

FISH studies were undertaken of the proband's genome to determine if the copy number changes that were identified by molecular karyotyping were due to a tandem duplication event. Locus-specific probes showed target hybridisation to the short arm of both homologues of chromosome 3, with an enhanced signal for both probes on the same homologue (Figure 2). These results suggest the presence of two tandem duplications in close proximity to one another, supporting the molecular karyotyping findings.

## 4. Discussion

The *CHL1* gene encodes a protein that is part of the L1 gene family of neural adhesion molecules that regulate brain cell migration and synaptogenesis [11]. It is highly expressed in the central and peripheral nervous systems. Our duplicated region includes three transcript variants; only the 5′ untranslated regions of transcript variants 1 and 2 are included in the duplicated segment. The segment of the *CNTN6* gene that is duplicated also corresponds to an untranslated region. The *CNTN6* gene encodes a neural adhesion molecule that is part of the immunoglobulin superfamily [20, 21]. This gene plays important roles in the formation, maintenance, and plasticity of functional neuronal networks in the central nervous system.

The proposed pathogenic mechanism for the 3p deletion syndrome is haploinsufficiency of several crucial genes [12, 22]. The more proximal genes on 3p (*CRBN* and *CNTN4*) are thought to account for the dysmorphic features [9] and mental retardation [10], while the distal gene, *CHL1*, may also be involved in impaired cognitive functioning [12, 13].

In the limited number of case studies with anomalies restricted to the *CHL1* gene (Figure 1), the dysmorphic features have been varied but there is usually some degree of cognitive impairment. In one of the families reported by Pohjola et al. [7], both the proband and his mother carried the same 1.1 Mb deletion, containing only the *CHL1* gene. The proband's clinical presentation included slow physical development, microcephaly, reticular hyperpigmentation of the skin, temper tantrums, and severe learning disabilities. His facial dysmorphic features included hypotelorism, low forehead, and a long, thin, and pointed nose. His growth parameters, apart from head circumference, were within normal ranges. His mother shared similar facial features, except for hypotelorism and microcephaly. Her growth and development were completely normal. The authors suggested that the atypical presentation of the proband could possibly indicate two separate syndromes: the 3p deletion responsible for the mild mental retardation, and the other features, including skin hyperpigmentation, caused by a distinct but unknown aetiology [7].

In the case report by Cuoco et al. [6], the terminal deletion carrying only the *CHL1* gene was transmitted from a normal father to two affected sons. Both sons had mild mental retardation characterized by learning and language difficulties, but not the distinct features of the 3p deletion phenotype. The growth parameters for both brothers were within normal ranges. The first son also had tonic-clonic seizures, the first at 8 years of age, and required therapy with a single agent. The father, carrying the same 3p deletion, had completed studies as a dentist and never had any physical impairment [6].

A third case study identified a man with nonspecific mental retardation carrying a translocation 46, Y, t(X;3)(p22.1;p26.3) [12]. His clinical presentation included overall bradykinesia, low mental level, bradyphrenia, poor attention span, and mild echolalia. The Xp breakpoint did not affect a known or predicted breakpoint so the phenotype was presumably caused by disruption of one allele of the *CHL1* gene alone. The second *CHL1* allele was sequenced, and no intragenic mutation was identified. This case also supports the pathogenic mechanism of haploinsufficiency of *CHL1* for nonspecific mental retardation. The authors went further to catalogue *Chl1* expression levels in mice hipoccampi. *Chl1* is the mouse ortholog of *CHL1* (close homolog of L1). The authors found that found that *Chl1* gene expression levels in the hippocampus of *Chl* ^*+/−*^ mice were half those found in wild type mice, but with normal through to abnormal behaviour.

In the first case study reporting microduplication in the *CHL1* gene, the entire gene was duplicated [13]. The phenotype of the girl in this study included intellectual disability. Her gross development progressed normally and she reached her first motor milestones within the normal timeframes. She showed marked speech development delay after two years. She also displayed paroxysmal eyelid myoclonia from 3 months to 3 years and had generalised tonic-clonic seizures that commenced in her second year of life and required dual therapy. She was followed until 16 years of age and at this stage still showed significant intellectual disabilities. The authors did not postulate any models of pathogenesis for *CHL1* gene duplication. They did suggest that the phenotypes for both microdeletion and microduplication were similar; however, they acknowledged that the number of reported patients was too low to claim this with confidence [13].

In addition, the correlation of genotype with phenotype is complicated by the observation that both deletion and duplication are associated with incomplete penetrance [13]. The two familial deletions of the *CHL1* gene [6, 7] and the duplication [13] were all transmitted by healthy parents. In *Chl* ^*+/−*^ mice, there is a spectrum from normal to abnormal behaviour [12]. This spectrum may arise from different genetic backgrounds of the mice used in these studies; hence, genetic factors that lie outside the CNVs identified in human case reports may be playing a role in observed phenotypic variability.

Small scale duplications and their phenotypic spectrum are diverse, widespread, and incompletely understood [23]. They are thought to contribute significantly to genomic variation both in creating phenotypic diversity and in some cases causing disease [23]. With dosage-sensitive genes, under- and overexpression phenotypes can give rise to the same phenotype, or different phenotypes. In the case of the former, the gene balance hypothesis suggests that under- and overexpression of genes encoding for proteins that comprise a multimeric regulatory protein complex disturb the stoichiometry of protein subunits and can lead to the same clinical phenotype even though the underlying molecular mechanism differs [23]. This is contradicted by the insufficient amount hypothesis, which suggests that haploinsufficient genes are required at abnormally high levels, so they are more sensitive to reductions than increases in dosage. This hypothesis explains the different phenotypes seen in under- and overexpression of some genes.

Unfortunately, the case reported here has several major phenotypic and genotypic differences compared to the previous *CHL1* duplication case so we are unable to confirm a clear phenotype that is distinct from patients carrying *CHL1* gene deletions. In the case of phenotype, motor delay, a key feature in this case, is not apparent in the previous microduplication case. As mentioned above, the previous case reached her first motor milestones within the normal range, and no lower limb spasticity was reported. Additionally, her onset of speech delay was later than our case. The previous study had the advantage of following the subject until 16 years of age, when she continued to show significant intellectual disabilities [13]. Unfortunately, as our case is under 3 years of age, intellectual assessment is incomplete.

The distal duplicated region in our case (only 104 kb involving the 5′ region of the *CHL1* gene) is smaller than the previously reported microduplication of 1.07 Mb that encompasses the entire *CHL1* gene [13]. The significance of duplicating the amino terminal regions of the *CHL1* and *CNTN6* genes is not known. It is possible that they affect the expression of their entire respective genes or even that of neighbouring genes. There are several proposed transcription factor binding sites upstream of the transcription start site of the *CHL1* gene that are contained within the duplicated segment [14]. Therefore, the duplication breakpoints flanking the amino terminal region of the *CHL1* gene may lead to gene disruption and hence reduced gene expression. In addition, there is a proposed 63 bp upstream open reading frame (uORF) that lies in the 5′ untranslated region of transcripts 1 and 2 of the *CHL1* gene [24]. uORFs have been suggested to regulate gene expression by largely reducing translational efficiency of the main ORF [25].

**Figure 3:**
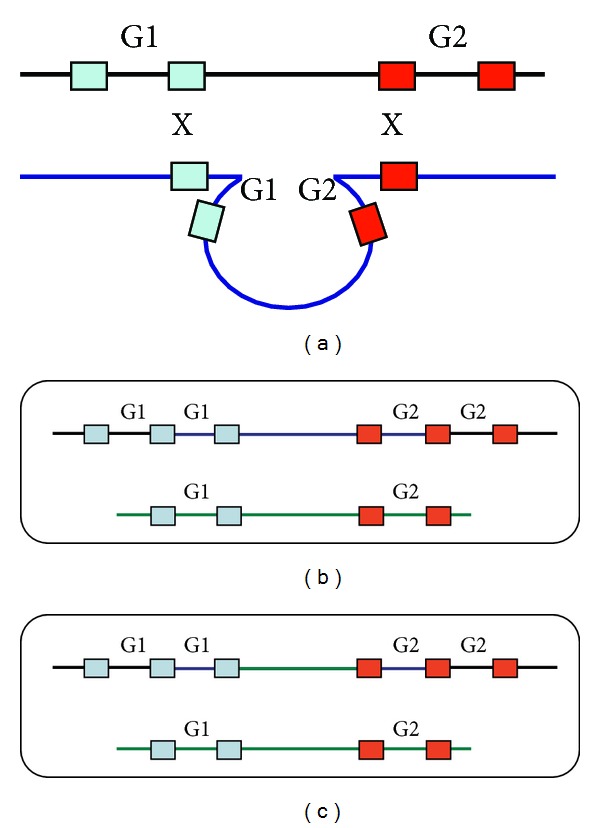
Schematic of hypothetical meiotic and mitotic recombination events leading to the observed allelic and copy number imbalances in the proband. (a) Shows a possible exchange between homologous chromosomes at meiosis that could lead to a copy number gain in two neighbouring genes (labelled G1 and G2); the coloured boxes represent flanking alleles of a repetitive sequence. (b) Shows the homologous chromosomes 3 of the proband at conception. (c) Shows a possible early mitotic event (between homologous copies of chromosome 3) during development of the proband that would give rise to interstitial segmental isodisomy in the region bounded by genes 1 and 2. The contribution of this cell line with that of the cell line represented in (b) would be consistent with the allelic imbalance and copy number data of the proband.

Another anomaly in our case is the area of copy-neutral allelic imbalance between the two duplicated regions. This region of approximately 220 kb contains the remainder of the *CHL1* gene but no other genes. A proposed mechanism for a copy-neutral allelic imbalance region flanked by duplicated regions could involve two separate events. The first is a meiotic recombination event leading to a duplication of the 3pter region.  Figure 3 shows a proposed mechanism of nonallelic homologous recombination involving repetitive elements flanking two genes. The maintenance of allelic imbalance in a copy-neutral region suggests two cell lines, both with the duplications present, but differing in the copy-neutral region. In one of the cell lines the region between the two duplications may have undergone interstitial segmental isodisomy due to an exchange between homologous chromosomes and retention of only one of the recombinant outcomes [26]. It is possible that the flanking duplicated regions predispose the intervening region of chromosome 3 to this exchange event.

## 5. Conclusions

As previously recognised, the number of patients with duplications of the *CHL1* gene is too small to classify this anomaly as definitely disease causing [13]. This case adds to the limited literature and is complicated by a more complex chromosomal imbalance compared to other reports.

In support of other cases, the proband reported here demonstrates complex rearrangements within 3p26.3 and its correlation with neurodevelopment. Again, our analysis highlights the importance of understanding the pathogenic mechanism of dosage-sensitive genes in their under- and overexpressed states, and in particular the *CHL1* gene, given its vital function in cognitive development. In this respect, family studies are ongoing in the case reported here.
